# Pattern II and pattern III MS are entities distinct from pattern I MS: evidence from cerebrospinal fluid analysis

**DOI:** 10.1186/s12974-017-0929-z

**Published:** 2017-08-29

**Authors:** S. Jarius, F.B. König, I. Metz, K. Ruprecht, F. Paul, W. Brück, B. Wildemann

**Affiliations:** 1 Molecular Neuroimmunology Group, Department of Neurology, University of Heidelberg, Heidelberg, Germany; 2 Department of Neuropathology, University of Göttingen, Göttingen, Germany; 3 Department of Neurology, Charité University Medicine Berlin, Berlin, Germany; 4 NeuroCure Clinical Research Center and Clinical and Experimental Multiple Sclerosis Research Center, Berlin, Germany

**Keywords:** Multiple sclerosis, Histopathology, Pattern I lesions, Pattern II lesions, Pattern III lesions, Cerebrospinal fluid, Oligoclonal bands, Intrathecal IgG synthesis, Blood-CSF barrier dysfunction, Total protein, QIgG, QAlb

## Abstract

**Background:**

The diagnosis of multiple sclerosis (MS) is currently based solely on clinical and magnetic resonance imaging features. However, histopathological studies have revealed four different patterns of lesion pathology in patients diagnosed with MS, suggesting that MS may be a pathologically heterogeneous syndrome rather than a single disease entity.

**Objective:**

The aim of this study was to investigate whether patients with pattern I MS differ from patients with pattern II or III MS with regard to cerebrospinal fluid (CSF) findings, especially with reference to intrathecal IgG synthesis, which is found in most patients with MS but is frequently missing in MS mimics such as aquaporin-4-IgG-positive neuromyelitis optica spectrum disorders and myelin oligodendrocyte glycoprotein-IgG-positive encephalomyelitis.

**Methods:**

Findings from 68 lumbar punctures in patients who underwent brain biopsy as part of their diagnostic work-up and who could be unequivocally classified as having pattern I, pattern II or pattern III MS were analysed retrospectively.

**Results:**

Oligoclonal bands (OCBs) were present in 88.2% of samples from pattern I MS patients but in only 27% of samples from patients with pattern II or pattern III MS (*P* < 0.00004); moreover, OCBs were present only transiently in some of the latter patients. A polyspecific intrathecal IgG response to measles, rubella and/or varicella zoster virus (so-called MRZ reaction) was previously reported in 60–80% of MS patients, but was absent in all pattern II or III MS patients tested (*P* < 0.00001 vs. previous cohorts). In contrast, the albumin CSF/serum ratio (QAlb), a marker of blood–CSF barrier function, was more frequently elevated in samples from pattern II and III MS patients (*P* < 0.002). Accordingly, QAlb values and albumin and total protein levels were higher in pattern II and III MS samples than in pattern I MS samples (*P* < 0.005, *P* < 0.009 and *P* < 0.006, respectively).

**Conclusions:**

Patients with pattern II or pattern III MS differ significantly from patients with pattern I MS as well as from previous, histologically non-classified MS cohorts with regard to both intrathecal IgG synthesis and blood–CSF barrier function. Our findings strongly corroborate the notion that pattern II and pattern III MS are entities distinct from pattern I MS.

## Background

Histopathological studies have demonstrated at least three different lesion patterns in early disease stages from patients diagnosed with multiple sclerosis (MS), termed patterns I, II and III [1, 2]. Pattern I lesions show T cell and macrophage infiltration. Pattern II is defined by additional antibody and complement deposition, suggesting a contribution of humoral mechanisms to disease pathology. Pattern III is characterized by distal oligodendrogliopathy with dysregulated myelin protein expression and oligodendrocyte apoptosis, but still occurs on an inflammatory background. A fourth pattern, defined by oligodendrocyte degeneration in the periplaque white matter, has been described in few autopsy cases of primary-progressive MS, but is rare. These findings raise the possibility that MS, a diagnosis currently based mainly on phenotypical, namely clinical and radiological features [3], may in fact be a pathologically heterogeneous syndrome rather than a single disease entity. Importantly, two recent studies demonstrated intraindividual homogeneity and persistence of pattern I, II and III lesions over time [4, 5], further corroborating the notion that lesion pathology may rather define pathogenetically distinct entities than reflect stage-dependent processes in the development of lesions.

Intrathecal IgG synthesis, as detected qualitatively by isoelectric focusing (IEF) of cerebrospinal fluid (CSF) and serum or quantitatively by calculation of the immunoglobulin CSF/serum ratio (QIgG), is present in 90–98% of MS patients, usually remains detectable over the entire course of disease and is considered a diagnostic mainstay in MS [6–9].

In this study, we retrospectively compared the CSF profiles of patients who underwent brain biopsy as part of their diagnostic work-up and who could be unequivocally classified as having pattern I, pattern II or pattern III lesions, respectively.

## Methods

### Patients

Results from 68 routine lumbar punctures in 33 patients with histopathologically confirmed MS (16 × pattern II, 7 × pattern III, 10 × pattern I [1, 2]) were analysed for OCB frequency and OCB patterns; CSF and serum IgG, IgM and IgA; CSF and serum albumin; IgG, IgM and IgA CSF/serum ratios (QIgG, QIgM, QIgA); albumin CSF/serum ratio (QAlb); intrathecal IgG response to measles (M), rubella (R) and varicella zoster (Z) (MRZ reaction [MRZR]) [10–14]; CSF total protein (TP) and CSF l-lactate levels; and CSF white cell counts and white cell differentiation. All patients had undergone brain biopsy as part of their diagnostic work-up. All biopsies were histopathologically classified at the Department of Neuropathology, University of Göttingen, Germany (WB, IM, FK), as previously described [1, 2]. All patients were classified based on brain lesions; none was classified based on brainstem, spinal cord or optic nerve lesions. All had early active disease and none had primary-progressive disease or pattern IV lesions. The median age at the time of first lumbar puncture was 36 years (range 13–63). The sex ratio (m:f) was 1:1.75. All patients were of Caucasian origin. Available serum samples were retrospectively tested for aquaporin 4 (AQP4)-IgG and myelin oligodendrocyte glycoprotein (MOG)-IgG using a cell-based assay (CBA) employing formalin HEK293 cells transfected with full-length human M1-AQP4 and M23-AQP4 [15, 16] or full-length human MOG [17, 18], respectively. All analyses were done retrospectively, in no case were brain, blood or CSF specimens obtained for the present study. AQP4-IgG and MOG-IgG testing and retrospective analysis of the patients’ CSF results was performed in an anonymized fashion.

### Evaluation of the humoral immune response

Oligoclonal IgG bands (OCBs) were evaluated according to an international consensus [9]: IEF pattern 1 = no OCBs in CSF or serum; IEF pattern 2 = CSF-restricted OCBs; IEF pattern 3 = CSF-restricted OCBs and additional identical bands in CSF and serum (combination of patterns 2 and 4); IEF pattern 4 = identical OCBs in CSF and serum (‘mirror pattern’); and IEF pattern 5 = monoclonal bands in CSF and serum. Only IEF patterns 2 and 3 indicate intrathecal IgG synthesis. Quantitative expressions of the intrathecal humoral immune response were based on calculation of the CSF/serum ratios for IgG (QIgG), IgM (QIgM) and IgA (QIgA) with Q_Ig_ = Ig_CSF[mg/L]_/Ig_serum[g/L]_. The upper limits of the respective reference ranges, Q_lim_(IgG), Q_lim_(IgM) and Q_lim_(IgA), were calculated against QAlb according to Reiber’s revised hyperbolic functions [10]. Values for Q_Ig_ > Q_lim_(Ig) were considered to indicate intrathecal immunoglobulin synthesis [10]. The fraction (in %) of intrathecally produced Ig (Ig_IF_) and the absolute amount of locally, i.e. intrathecally, produced Ig (IgG_loc_) were calculated according to the following formulas: Ig_IF[%]_ = [QIg − Q_lim_(Ig)] × Ig_serum_ × 100 and Ig_loc[mg/L]_ = [QIg − Q_lim_(Ig)] × Ig_serum_, respectively [10]. Antibody indices (AI) were calculated according to Reiber’s formula: AI = Q_spec_/QIgG, or AI = Q_spec_/Q_lim_(IgG) if (Q_lim_ > QIgG), with Q_spec_ = IgG_spec_(CSF)/IgG_spec_(serum). Taking into account the relatively small sample size of the pattern I MS subgroup, we also compared the results for pattern II and pattern III MS with data from ‘classical’ landmark studies on OCB and MRZR findings in (histologically non-classified) MS [14, 19].

### Evaluation of blood-CSF barrier function

The CSF/serum albumin quotient, QAlb = Alb_CSF[mg/L]_/Alb_serum[g/L]_, was used to assess the blood*–*CSF barrier function. As the upper reference limit of QAlb is age dependent, Q_lim_(Alb) was calculated as 4 + (*a*/15), with *a* representing the patient’s age, according to Reiber et al. [20]. Dysfunction of the blood–CSF barrier was defined as QAlb > Q_lim_(Alb).

### Cytological examination, total CSF protein and l-lactate

A white cell count >5/μL was classified as ‘increased’. An age-dependent upper reference range for CSF l-lactate was applied (0–15 years of age, 1.8 mmol/L; 16–50 years, 2.1 mmol/L; >50 years, 2.6 mmol/L [21, 22]). As upper reference limit for total CSF protein, 450 mg/L was used.

### Statistics

Fisher’s exact test and Mann*–*Whitney *U* test were used to detect differences between groups. Spearman’s rho was calculated to test for correlations. *P* values <0.05 were considered statistically significant. Due to the exploratory nature of this study, no corrections for multiple comparisons were performed. Reiber diagrams were generated using the *Protein Statistics in CSF analysis V3.0* software (Comed, Soest, Germany). The study was approved by the institutional review boards of the University of Göttingen and the University of Heidelberg. All CSF parameters evaluated in this study are routinely tested in Germany as part of the diagnostic workup of patients with suspected MS in Germany and are recommended by the guidelines of the German Society of Neurology and by the guidelines of the Germany Society of CSF Analysis and Clinical Neurochemistry [21].

## Results

### Epidemiology and autoantibody status

Epidemiological data for all subgroups are given in Table 1. All serum samples available for retrospective testing (*n* = 13) were negative for AQP4-IgG and MOG-IgG [18].

**Table 1:** Epidemiological findings

	Units	Pattern I MS	Patterns II + III MS﻿	Pattern II MS﻿	Pattern III MS﻿	AQP4-Ab+ NMO [48]
Patients	*N*	10	23	16	7	89
Samples	*N*	19	49	30	19	211
Age at first LP	Years	30 (13–47)	36 (20–63)	35 (20–51)	38 (26–63)	39.5 (14–79)
Sex ratio	m:f	1:1	1:2.3	1:2.2	1:2.5	1:12.1

*LP* lumbar puncture, *m* male, *f* female

Results in AQP4-IgG-positive NMO as observed in a previous study [48] are given in the last column for comparison. Note the marked difference in the sex ratios between pattern II MS and NMO. Years are given as median and range

### Oligoclonal bands

**Fig. 1:**
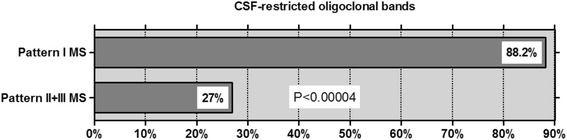
Frequency of CSF-restricted oligoclonal bands (OCBs) results from 54 LPs in patients with pattern I MS compared to patients with pattern II or pattern III MS. Note that OCBs were present only transiently in 2/7 OCB-positive pattern II/III patients

CSF-restricted OCB were found in 15/17 (88.2%) samples from patients with pattern I MS, but were negative in 27/37 (73%) of samples from patients with pattern II or pattern III MS (*P* < 0.00004) (Fig. 1). Overall, only 7/22 (31.8%) pattern II and III patients, but 8/10 (80%) pattern I MS patients, had OCB at least once (*P* < 0.021). Moreover, OCBs were only transiently positive in 2 of the 7 only OCB-positive pattern II and III patients (patient 1, pattern II: lumbar puncture (LP) #1 positive, LP #2 negative; patient 2, pattern III: LP #1 positive, LP #2 negative). If only persisting OCB are taken into account, only 22.7% (5/22) patients with pattern II or III MS were positive for OCB (*P* < 0.006) (Table 2). Of note, QIgG was negative at the time of OCB determination in the two cases with transient OCB, indicating very low levels of intrathecal IgG synthesis in these patients. Identical OCB in serum and CSF without additional CSF-restricted bands (the so-called mirror pattern or IEF pattern 4), suggesting possible systemic inflammation, were found in a single patient with pattern II MS but were absent in all other patients. IEF pattern 5, indicating monoclonal gammopathy, was present in a single pattern II MS patient (still detectable in a second sample taken 24 months later) and transiently in one pattern III patient (negative follow-up sample obtained 36 days later). Comparison of the pattern II or pattern III MS patients’ data with data from a reference paper on CSF in MS [19] confirmed the marked difference in OCB frequency (*P* < 0.000001) (see Table 9 for details).

**Table 2:** Frequency of intrathecal total IgG synthesis; oligoclonal IgG pattern; IgG CSF/serum ratios; intrathecal IgG fractions; absolute amount of locally produced IgG; and CSF and serum IgG concentrations

	Units	Pattern I MS	Pattern II + III MS	Pattern II MS	Pattern III MS	AQP4-IgG+ NMO [48]	MOG-IgG+ EM [35]
CSF-restricted OCB	Samples	15/17 (88.2)	10/37 (27)	9/25 (36)	1/12 (8.3)	29/177 (16.4)	8/47 (17)
CSF-restricted OCB	Patients	8/10 (80)	7/22 (31.8)	6/16 (37.5)	1/6 (16.7)	22/80 (27.5)	6/45 (13)
CSF-restricted OCB, without transient OCB	Patients	8/10 (80)	5/22 (22.7)	5/16 (31.3)	0/6 (0)	16/80 (20)	6/45 (13)
OCB, IEF pattern 1	Samples	2/15 (13.3)	24/35 (68.6)	13/23 (56.5)	11/12 (91.7)	127/177 (71.8)	n.d.
OCB, IEF pattern 2	Samples	13/15 (86.7)	7/35 (20)	6/23 (26.1)	1/12 (8.3)	19/177 (10.7)	n.d.
OCB, IEF pattern 3	Samples	0/15 (0)	1/35 (2.9)	1/23 (4.3)	0/12 (0)	10/177 (5.6)	n.d.
OCB, IEF pattern 4	Samples	0/15 (0)	1/35 (2.9)	1/23 (4.3)	0/12 (0)	20/177 (11.3)	n.d.
OCB, IEF pattern 5	Samples	0/15 (0)	3/35 (8.6)	2/23 (8.7)	1/12 (8.3)	1/177 (0.6)	n.d.
QIgG > Q_lim_(IgG)	Patients	3/10 (30)	4/21 (19)	4/14 (28.6)	0/7 (0)	n.d.	n.d.
QIgG > Q_lim_(IgG)	Samples	7/17 (41.2)	5/41 (12.2)	5/24 (20.8)	0/17 (0)	13/143 (9)	n.d.
QIgG, all LPs	–	3.6 (1.9–9.35; 17)	4.75 (1.3–21.5; 42)	4.8 (1.3–21.5; 25)	4.7 (1.98–12.6; 17)	3.9 (0.2–58.1; 135)	n.d.
QIgG, if positive	–	3.63 (2.6–9.35; 7)	5.03 (3.2–8.8; 5)	5.03 (3.2–8.8; 5)	n.a.	5.7 (2.5–58.1; 12)	n.d.
IgG_loc_, all LPs	mg/L	0 (0–75.76; 17)	0 (0–51.17; 41)	0 (0–51.17; 24)	0 (0–0; 17)	0 (0–441.4; 135)	n.d.
IgG_loc_, QIgG positives	mg/L	5.91 (2.31–75.76; 7)	16.75 (11.79–51.17; 5)	16.75 (11.79–51.17; 5)	n.a.	13.9 (1.3–441.4; 12)	n.d.
IgG_IF_, all LPs	%	0 (0–0.58; 17)	0 (0–0.59; 41)	0 (0–0.59; 24)	0 (0–0; 17)	0 (0–90.6; 135)	n.d.
IgG_IF_, QIgG positives	%	0.14 (0.06–0.58; 7)	0.47 (0.22–0.59; 5)	0.47 (0.22–0.59; 5)	n.a.	32.5 (2.8–90.6; 12)	n.d.
IgG CSF, all LPs	mg/L	39.9 (11–130; 17)	43.7 (11.4–146; 41)	46 (11.4–109; 24)	37.7 (21–146; 17)	37.9 (2.4–487; 136)	n.d.
IgG CSF, QIgG positives	mg/L	39.9 (16.8–130; 7)	53.3 (22.9–109; 5)	53.3 (22.9–109; 5)	n.a.	70.6 (20.5–487; 12)	n.d.
IgG serum, all LPs	g/L	10 (3.9–28.04; 17)	8.75 (5.55–28.9; 41)	10.39 (5.55–24.8; 24)	7.5 (6.15–28.9; 17)	10 (1.9–55.8; 134)	n.d.
IgG serum, QIgG positives	g/L	9.21 (3.9–15.3; 7)	8.75 (7.1–12.4; 5)	8.75 (7.1–12.4; 5)	n.a.	10.7 (2.4–18; 12)	n.d.

*n.a.* not applicable, *n.d.* no data

Concentrations, ratios and fractions are reported as medians; range and total sample numbers examined are given in brackets

### IgG, IgM and IgA CSF/serum ratios

**Fig. 2:**
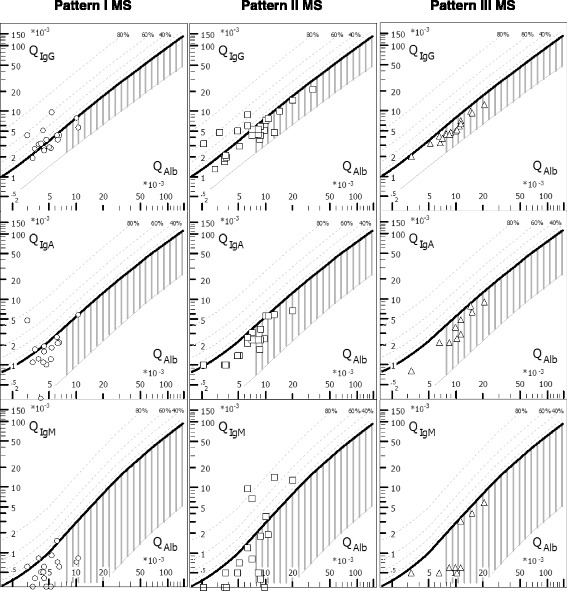
CSF/serum quotient diagrams for IgG, IgM and IgA (‘Reibergrams’). Individual CSF/serum ratios of IgG, IgA and IgM are plotted against CSF/serum albumin ratios. Values above the upper hyperbolic discrimination line, Q_lim_, indicate intrathecal synthesis of the respective immunoglobulin (Ig) class. Individual intrathecal fractions, Ig_IF_, can be directly read by interpolation from the percentiles above Q_lim_ (median values are given in Tables 2 and 3). *Vertical dashed lines* indicate the median Q_lim_(Alb). *IgG/A/M* immunoglobulin G/A/M, *QIgG/A/M* CSF/serum IgG/A/M ratios, *QAlb* CSF/serum albumin ratio

While 7/17 (41.2) samples from patients with pattern I MS showed elevated QIgG levels, only 5/41 (12.2) of samples from patients with pattern II or pattern III MS did so (*P* < 0.021) (Table 2). Intrathecal production of IgM as indicated by elevated QIgM was rare and was found both in patients with pattern I MS (3/9; 33.3%) and in patients with pattern II MS (3/12; 25%). QIgA levels were elevated in a few pattern I patients (2/8; 25%), in a single pattern II patient and in none of the pattern III patients (1/17; 5.9%). Data on both the fractions and the absolute amounts of intrathecally produced IgG, IgM and IgA can be found in Tables 2 and 3. Plots of QIgG, QIgA and QIgM, respectively, against QAlb as a measure of blood–CSF barrier function are shown in Fig. 2.

**Table 3:** Frequency of intrathecal IgM and IgA synthesis; IgM and IgA CSF/serum ratios; intrathecal IgM and IgA fractions; amount of locally produced IgM and IgA; and absolute CSF and serum IgM and IgA concentrations

	Units	Pattern I MS	Pattern II + III MS	Pattern II MS	Pattern III MS	AQP4-IgG+ NMO [48]
QIgM > Q_lim_(IgM)	Patients	3/9 (33.3)	3/17 (17.6)	3/12 (25)	0/5 (0)	n.d.
QIgM > Q_lim_(IgM), LPs	Samples	3/16 (18.8)	5/30 (16.7)	5/20 (25)	0/10 (0)	13/96 (14%)
QIgM, all LPs	–	0.58 (0.26–1.5; 15)	0.65 (0.1–14.1; 26)	0.8 (0.1–14.1; 17)	0.6 (0.5–5.8; 9)	0.9 (0–40.5; 97)
QIgM, if positive	–	0.8 (0.58–1.5; 3)	9.8 (3.5–14.1; 5)	9.8 (3.5–14.1; 5)	n.a.	8.3 (1.2–40.5; 13)
IgM_loc_, all LPs	mg/L	0 (0–0.27; 16)	0 (0–9.24; 29)	0 (0–9.24; 19)	0 (0–0; 10)	0 (0–35.3; 95)
IgM_loc_, QIgM positives	mg/L	0.12 (0.05–0.27; 3)	6.41 (0.86–9.24; 5)	6.41 (0.86–9.24; 5)	n.a.	0.9 (0–35.3; 13)
IgM_IF_, all LPs	%	0 (0–0.12; 16)	0 (0–0.86; 30)	0 (0–0.86; 20)	0 (0–0; 10)	0 (0-95.8; 95)
IgM_IF_, QIgM positives	%	0.1 (0.09-0.12; 3)	0.71 (0.17–0.86; 5)	0.71 (0.17–0.86; 5)	n.a.	15.3 (2.3–95.8; 13)
IgM CSF, all LPs	mg/L	1 (0.4–3; 15)	1.05 (0.2–17.8; 26)	1.2 (0.2–17.8; 17)	0.9 (0.54–11; 9)	0.7 (0–36.9; 100)
IgM CSF, QIgM positives	mg/L	1.2 (0.42–3; 3)	9 (5.4–17.8; 5)	9 (5.4–17.8; 5)	n.a.	n.d.
IgM serum, all LPs	g/L	1.68 (0.56–3.75; 16)	1.6 (0.38–3.68; 29)	1.45 (0.38–3.68; 20)	1.7 (0.9–2.2; 9)	0.96 (0.13–2.85; 99)
IgM serum, QIgM positives	g/L	1.51 (0.72–2; 3)	1.1 (0.64–1.5; 5)	1.1 (0.64–1.5; 5)	n.a.	n.d.
QIgA > Q_lim_(IgA)	Patients	2/8 (25)	1/17 (5.9)	1/12 (8.3)	0/5 (0)	n.d.
QIgA > Q_lim_(IgA)	Samples	2/15 (13.3)	1/29 (3.4)	1/19 (5.3)	0/10 (0)	5/88 (6%)
QIgA, all LPs	–	1.77 (1.01–5.7; 13)	2.5 (0.82–9.1; 29)	2.4 (1–6.6; 19)	3.3 (0.82–9.1; 10)	2.1 (0–40.4; 88)
QIgA, if positive	–	3.22 (1.73–4.7; 2)	1 (1–1; 1)	1 (1–1; 1)	n.a.	5.1 (3.7–20.2; 5)
IgA_loc_, all LPs	mg/L	0 (0–3.72; 15)	0 (0–0.08; 29)	0 (0–0.08; 19)	0 (0–0; 10)	0 (0–3.2; 86)
IgA_loc_, QIgA positives	mg/L	2.015 (0.31–3.72; 2)	0.08 (0.08–0.08; 1)	0.08 (0.08–0.08; 1)	n.a.	0.3 (0.03–3.2; 5)
IgA_IF_, all LPs	%	0 (0–0.73; 15)	0 (0–0.08; 29)	0 (0–0.08; 19)	0 (0–0; 10)	0 (0–7.5; 86)
IgA_IF_, QIgA positives	%	0.425 (0.12–0.73; 2)	0.08 (0.08–0.08; 1)	0.08 (0.08–0.08; 1)	n.a.	5.6 (0.5–7.5; 5)
IgA CSF, all LPs	mg/L	2.7 (1.4–16.6; 13)	4.55 (0.24–19.9; 30)	3.45 (0.24–17.5; 20)	8.5 (3.1–19.9; 10)	4.7 (0–131; 95)
IgA CSF, QIgA positives	mg/L	3.79 (2.5–5.08; 2)	1.1 (1.1–1.1; 1)	1.1 (1.1–1.1; 1)	n.a.	n.d.
IgA serum, all LPs	g/L	1.53 (0.9–2.9; 15)	1.88 (0.53–3.8; 29)	1.55 (0.53–3; 19)	2.6 (1.2–3.8; 10)	0.96 (0.13–2.85; 99)
IgA serum, QIgA positives	g/L	1.26 (1.08–1.45; 2)	1.1 (1.1–1.1; 1)	1.1 (1.1–1.1; 1)	n.a.	n.d.

*n*.*a*. not applicable, *n*.*d*. no data

Concentrations, ratios and fractions are reported as medians; range and total sample numbers examined are given in brackets

### Immunoglobulin class patterns

Intrathecal Ig production, if present, was restricted to one immunoglobulin class in most cases (cf. Table 4 for details). A two-class immune reaction, defined as intrathecal production of either IgG and IgM, or IgM and IgA, or IgG and IgA, was present in only four samples (Table 4). None of the samples showed a three-class immune response, defined as combined elevation of QIgG, QIgM and QIgA. Of note, three pattern II patients showed an isolated IgM reaction at least once (patient 1, intrathecal IgM, IgG and IgA fractions, 83, 47 and 0%, respectively, at first lumbar puncture; 73, 0 and 0% 10 days later; and 71, 0 and 0% 14 days later; patient 2, 37, 0 and 0%; patient 3, 17, 0 and 0% at first puncture, no intrathecal IgM, IgG and IgA synthesis at repeat puncture at day 34 and day 815).

**Table 4:** Immunoglobulin class response patterns

	Units	Pattern I MS	Pattern II + III MS	Pattern II MS	Pattern III MS	AQP4-IgG+ NMO [48]
Three-class reaction	Samples	0/16 (0)	0/32 (0)	0/21 (0)	0/11 (0)	0/87 (0%)
Two-class reaction	Samples	2/16 (12.5)	2/32 (6.3)	2/21 (9.5)	0/11 (0)	5/87 (5.7%)
IgG + IgM	Samples	1	1	1	0	1/87 (1.1%)
IgG + IgA	Samples	1	1	1	0	1/87 (1.1%)
IgM + IgA	Samples	0	0	0	0	3/87 (3.4%)
One-class reaction	Samples	5/16 (31.3)	6/32 (18.8)	6/21 (28.6)	0/11 (0)	13/87 (14.9%)
Only IgG	Samples	4	2	2	0	5/87 (5.7%)
Only IgM	Samples	1	4	4	0	7/87 (8%)
Only IgA	Samples	0	0	0	0	1/87 (1.1%)

Concentrations, ratios and fractions are reported as medians; range and total sample numbers examined are given in brackets

QIgG/A/M=CSF/serum IgG/A/M ratios

### MRZ reaction

A positive MRZR, as defined by at least two positive IgG-AIs, which has been reported in the literature to be present in around 70% of patients with MS [10–14, 23], was absent in all samples from patients with pattern II or pattern III MS examined (*P* < 0.000002 compared to unselected MS patients [23]). Only two patients with pattern I MS had been tested for MRZR (positive in 1/2, 50%), precluding statistical comparisons. A positive AI against at least one of the three constituents (measles virus, rubella virus, varicella zoster virus), which has been reported in the literature to be present in 89% (158/177) [19] to 94% (94/100) [14] of patients with MS, was absent in 91% (10/11) of samples from pattern II and III patients (*P* < 0.000001 vs. references [19] and [14]). MRZR was re-tested in two patients (one with pattern II and one with pattern III MS) later in the disease course and remained negative in both cases. Only a single patient with pattern III MS had a positive AI for measles virus; however, a follow-up examination 1 month later was negative. See Table 5 for details.

**Table 5:** Frequency of intrathecal IgG synthesis to infectious agents

	Units	MS, according to literature [14]	Pattern I MS	Pattern II + III MS	Pattern II MS	Pattern III MS	AQP4-IgG+ NMO [13, 23, 43, 55, 56]	MOG-IgG+ EM [35]
MRZ reaction (≥2 AIs >1.5)	Patients	72/100 (72)	1/2 (50)	0/10 (0)	0/7 (0)	0/3 (0)	1/42 (2.4)	0/11 (0)
	Samples	72/100 (72)	1/2 (50)	0/12 (0)	0/8 (0)	0/4 (0)	1/42 (2.4)	0/11 (0)
M and/or R and/or Z >1.5	Samples	158/177 (89)	1/2 (50)	1/11 (9.1)	0/8 (0)	1/3 (33.3)	n.d.	n.d.
Measles virus AI >1.5	Samples	138/177 (78)	1/2 (50)	1/10 (10)	0/8 (0)	1/4 (25)	1/42 (2.4)	0/11 (0)
Rubella virus AI >1.5	Samples	106/177 (60)	1/2 (50)	0/9 (0)	0/8 (0)	0/4 (0)	0/42 (0)	0/11 (0)
Zoster virus AI >1.5	Samples	97/177 (55)	0/2 (0)	0/9 (0)	0/8 (0)	0/4 (0)	1/42 (2.4)	0/11 (0)
Herpes simplex virus AI >1.5	Samples	26/94 (28)	n.d.	0/4 (0)	0/4 (0)	0/1 (0)	n.d.	n.d.
Epstein–Barr virus AI >1.5	Samples	n.d.	0/2 (0)	0/2 (0)	n.d.	n.d.	n.d.	0/2 (0)
*Borrelia burgdorferi* AI >1.5	Samples	0/1 (0)	0/3 (0)	0/6 (0)	0/2 (0)	n.d.	0/1 (0)	0/3 (0)
*Toxoplasma gondii* AI >1.5	Samples	n.d.	0/1 (0)	0/1 (0)	n.d.	n.d.	n.d.	0/1 (0)
TPHA AI >1.5	Samples	n.d.	0/1 (0)	0/1 (0)	n.d.	n.d.	n.d.	0/1 (0)

*n.a.* not applicable *AI* antibody index, *M* measles virus, *R* rubella virus, *TPHA Treponema pallidum* haemagglutination assay, *Z* varicella zoster virus

### Blood–CSF barrier integrity

A disrupted blood–CSF barrier function was found in only 4/17 (23.5) of samples from patients with pattern I MS but in 30/43 (69.8) of samples from patients with pattern II or pattern III MS (*P* < 0.002). Accordingly, median QAlb values and median albumin CSF concentrations were higher in pattern II or III samples than in pattern I samples (*P* < 0.005 for QAlb, *P* < 0.009 for CSF albumin). See Table 6 for details.

**Table 6:** Blood–CSF barrier function and albumin levels

	Units	Pattern I MS	Pattern II + III MS	Pattern II MS	Pattern III MS	AQP4-IgG+ NMO [48]
QAlb > Q_lim_(Alb)	Patients	4/10 (40)	15/22 (68.2)	9/15 (60)	6/7 (85.7)	n.d.
QAlb > Q_lim_(Alb)	Samples	4/17 (23.5)	30/43 (69.8)	16/26 (61.5)	14/17 (82.4)	75/147 (51%)
QAlb, all LPs	–	4.81 (3.01–10.8; 17)	8.39 (2.1–33.9; 42)	7.3 (2.1–33.9; 25)	9 (3.3–20.7; 17)	7 (2.3–57.1; 137)
QAlb, QAlb pos.	–	8.53 (6.3–10.8; 4)	9.8 (6.4–33.9; 29)	9 (6.4–33.9; 15)	10.25 (6.8––20.7; 14)	11.8 (5.63–57.14; 70)
Albumin CSF, all LPs	mg/L	204 (95–517; 17)	331 (81.2–726; 41)	255.5 (81.2–599; 24)	368 (137–726; 17)	284 (83.6–1890; 139)
Albumin CSF, QAlb pos.	mg/L	366 (251–517; 4)	371.5 (227–726; 28)	312 (227–599; 14)	431 (282–726; 14)	437 (219–1890)
Albumin serum, all LPs	g/L	40.6 (29.3–49.5; 17)	40 (22.6–51.8; 41)	39.85 (22.6–51.4; 24)	41.2 (32.7–51.8; 17)	40.7 (19.7–67.9; 133)
Albumin serum, QAlb pos.	g/L	43.15 (37.7–47.9; 4)	39.5 (22.6–50.9; 27)	35.55 (22.6–46.8; 14)	39.9 (32.7–50.9; 13)	39 (19.7–55.9)
Combined intrathecal IgG synthesis and disturbed blood–CSF barrier function						
Patients, at least once	*N* (%)	3/10 (30)	4/20 (20)	3/13 (23.1)	1/7 (14.3)	n.d.
All LPs	*N* (%)	3/17 (17.6)	5/43 (11.6)	4/26 (15.4)	1/17 (5.9)	13/74 (17.6%)

*n.d.* no data

QAlb = CSF/serum albumin ratio. Concentrations and ratios are reported as medians; range and total sample numbers examined are given in brackets

### Cellular immune response

CSF pleocytosis was slightly less frequent in pattern II MS samples (6/26; 23.1%) than in pattern I (5/13; 38.5%) and pattern III (10/14; 71.4%) samples (*P* < 0.02). However, median CSF white cell count (WCC) were low in all three subgroups, with only 3/49 (6.1%) of samples showing cell counts >40 cells/μL (see Table 7 for details). In patients with pleocytosis, CSF white cells included lymphocytes in all samples examined and monocytes in most, with no significant differences between the groups. Neutrophil granulocytes were present only in five samples from patients with pleocytosis, and the levels were very low (1 × pattern I MS: 7 cells/μL, 5% neutrophils; 1 × pattern II MS: 6 cells/μL, 3% neutrophils; 3 × pattern III MS: 17, 13 and 7 cells/μL, 1–3% neutrophils) and possibly related to slight blood contamination in one case (722 erythrocytes/μL). Eosinophils were present in only two patients with pleocytosis (1 × pattern II MS: 69 cells/μL, 13% eosinophils, absent both in a previous sample and in a follow-up sample, AQP4-IgG: negative; 1 × pattern III MS: 7 cells/μL, 5% eosinophils; no erythrocytes in both cases). Sixty-two percent (13/21) of pattern II/III MS samples without pleocytosis showed an elevated QAlb and, thus, an albuminocytological dissociation, but only 12.5% (1/8) of pattern I MS samples (P < 0.04; see Table 7 for details).

**Table 7:** CSF white cell counts

	Units	Pattern I MS	Pattern II + III MS	Pattern II MS	Pattern III MS	AQP4-IgG+ NMO [48]
Pleocytosis	Samples	5/13 (38.5)	16/40 (40)	6/26 (23.1)	10/14 (71.4)	98/194 (50.5)
WCC, all LPs	Cells/μL	5 (1–23; 13)	3 (0–267; 40)	2 (0–267; 26)	8.5 (3–24; 14)	6 (0–380; 182)
WCC, if elevated	Cells/μL	11 (6–23; 5)	14.5 (6–267; 16)	40 (6–267; 6)	10.5 (7–24; 10)	19 (6–380; 98)
WCC, >40 cells/μL	Samples	0/13 (0)	3/36 (8.3)	3/22 (13.6)	0/14 (0)	28/194 (14.4)
WCC, >40 cells/μL	Cells/μL	n.a.	69 (55–267; 3)	69 (55–267; 3)	n.a.	94 (43–380; 28)
Albuminocytological dissociation	Samples	1/8 (12.5)	13/21 (61.9)	10/18 (55.6)	3/3 (100)	27/75 (33.3)

*n.a.* not applicable

WCC = white cell count

WCC in the various groups are reported as medians; range and total sample numbers examined are given in brackets

### Total CSF protein

Total protein (TP) in the CSF was more frequently elevated in samples from patients with pattern II or III MS samples (29/43; 67.4%) than in pattern I MS (3/15; 20%) (*P* < 0.003). Accordingly, median TP CSF levels were higher in pattern II or III MS samples (median 550 mg/dl, range 168–1930) than in pattern I MS samples (median 375, range 187–750) (*P* < 0.006), with the highest levels detected in pattern III samples (median 640, range 260–1277). TP CSF levels >750 mg/dl were present in 8/43 (18.6%) samples from patients with pattern II or III MS, but were not detected in any of the pattern I MS patients. As expected, TP CSF and QAlb were strongly correlated both in the total cohort and within each of the three subgroups (*P* < 0.0001).

### CSF l-lactate

A trend towards higher median l-lactate level was noted in pattern II and III MS (1.9 mmol/L, range 0.8–3.3) compared with pattern I MS (1.3 mmol/L, range 1.1–2.35). Similarly, a higher proportion of patients with pattern II or III MS than patients with pattern I MS had elevated l-lactate levels at least once, but the difference was not statistically significant. See Table 8 for details.

**Table 8:** CSF total protein and CSF l-lactate

	Units	Pattern I MS	Pattern II + III MS	Pattern II MS	Pattern III MS	AQP4-IgG+ NMO [48]
CSF TP elevated	Patients	3/9 (33.3)	14/20 (70)	8/13 (61.5)	6/7 (85.7)	n.d.
CSF TP elevated	Samples	3/15 (20)	29/43 (67.4)	15/26 (57.7)	14/17 (82.4)	80/152 (52.6)
CSF TP, all LPs	mg/L	375 (187–750; 15)	550 (168–1930; 43)	501 (168–1930; 26)	640 (260–1277; 17)	473 (198–3620; 147)
CSF TP, if elevated	mg/L	724 (532–750; 3)	622 (470–1930; 29)	571 (471–1930; 15)	693 (470–1277; 14)	780 (45.4–3620; 68)
CSF TP, >750 mg/L	mg/L	0/15 (0)	8/43 (18.6)	4/26 (15.4)	4/17 (23.5)	41/147 (28)
CSF lactate elevated	Patients	2/8 (25)	6/14 (42.9)	5/11 (45.5)	1/3 (33.3)	n.d.
CSF lactate elevated	Samples	2/9 (22.2)	7/28 (25)	6/20 (30)	1/8 (12.5)	27/83 (32.5)
CSF lactate, all LPs	mmol/L	1.3 (1.1–2.35; 11)	1.9 (0.8–3.3; 28)	1.85 (0.8–3.3; 20)	1.9 (1.36–2.9; 8)	1.97 (0.87–6.8; 80)
CSF lactate, if elevated	mmol/L	2.175 (2–2.35; 2)	2.5 (2.2–3.3; 7)	2.4 (2.2–3.3; 6)	2.9 (2.9–2.9; 1)	2.9 (2.1–6.8; 27)
CSF lactate, >3 mmol/L	mmol/L	0/11 (0)	1/28 (3.6)	1/20 (5)	0/8 (0)	27/80 (33.8)

*n.d.* no data

Concentrations are reported as medians; range and total sample numbers examined are given in brackets

### Abnormal vs. normal results

Overall, 65/68 (95.6%) LPs showed some abnormality (either intrathecal IgG, IgM or IgA synthesis; disturbed blood–CSF barrier function; or elevated WCC, TP or l-lactate levels); the only three samples with normal results were all from patients with pattern II MS.

A summary of the statistically significant differences between groups can be found in Table 9.

**Table 9:** Summary of differences in CSF parameters between various MS subgroups as observed in the present study

Parameter	Diagnostic groups	Results	*P* values
CSF-restricted OCB, all LPs	Pattern I	15/17 (88.2)	*P* < 0.00004
	Patterns II + III	10/37 (27)	
	Reiber et al. [19]	262/267 (98)	*P* < 0.000001
	Patterns II + III	10/37 (27)	
	Pattern I	15/17 (88.2)	*P* < 0.0001
	Pattern II	9/25 (36)	
	Pattern III	1/12 (8.3)	
CSF-restricted OCB, patients	Pattern I	8/10 (80)	*P* < 0.03
	Patterns II + III	7/22 (31.8)	
	Reiber et al. [19]	262/267 (98)	*P* < 0.000001
	Patterns II + III	7/22 (31.8)	
CSF-restricted OCB, patients, w/o transient OCBs	Pattern I	8/10 (80)	*P* < 0.006
	Patterns II + III	5/22 (22.7)	
	Reiber et al. [19]	262/267 (98)	*P* < 0.00001
	Patterns II + III	5/22 (22.7)	
MRZ reaction (≥2 AIs >1.5), all LPs	Jarius et al. [23]	397/546 (69)	*P* < 0.000001
	Patterns II + III	0/12 (0)	
M and/or R and/or Z >1.5, all LPs	Reiber et al. [19]	158/177 (89)	*P* < 0.000001
	Patterns II + III	0/12 (0)	
QIgG > Q_lim_(lgG), all LPs	Pattern I	7/17 (41.2)	*P* < 0.03
	Patterns II + III	5/41 (12.2)	
QAlb > Q_lim_(Alb), all LPs	Pattern I	4/17 (23.5)	*P* < 0.002
	Patterns II + III	30/43 (69.8)	
	Pattern I	4/17 (23.5)	*P* = 0.002
	Pattern II	16/26 (61.5)	
	Pattern III	14/17 (82.4)	
QAlb, all LPs	Pattern I	4.81 (3.01–10.8; 17)	*P* < 0.005
	Patterns II + III	8.39 (2.1–33.9; 42)	
Albumin CSF, all LPs	Pattern I	204 (95–517; 17)	*P* < 0.009
	Patterns II + III	331 (81.2–726; 41)	
Total CSF protein elevated, all LPs	Pattern I	3/15 (20)	*P* < 0.003
	Patterns II + III	29/43 (67.4)	
Total CSF protein, all LPs	Pattern I	375 (187–750; 15)	*P* < 0.007
	Patterns II + III	550 (168–1930; 43)	
Albuminocytological dissociation, all LPs	Pattern I	1/8 (12.5)	*P* < 0.04
	Patterns II + III	13/21 (61.9)	

## Discussion

In this study, we systematically analysed the results from 68 lumbar punctures in patients who were histopathologically diagnosed with pattern I, pattern II or pattern III MS according to previously published criteria [2]. The most striking finding was the lack of intrathecal IgG synthesis in the vast majority of samples from pattern II or pattern III MS patients. Intrathecal IgG synthesis is considered a diagnostic mainstay of MS. While previous studies reported a frequency of OCB in MS of 90–98% in European patients, 73% of samples from pattern II or pattern III MS patients, including all pattern III patients, were negative in the present European cohort. Moreover, OCBs were positive only transiently in the only two patients with non-pattern I lesions and follow-up data. Transient OCBs have been reported in other neuroinflammatory diseases such as neuromyelitis optica (NMO), acute disseminated encephalomyelitis or CNS infection, whereas OCBs are thought to persist over the entire disease course of MS [24, 25]. Similarly, the polyspecific, intrathecal antiviral IgG response as defined by at least two elevated CSF/serum antibody indices to measles, rubella and/or (so-called MRZ reaction) varicella zoster virus, which is seen in around 70% of patients with MS (and considered to represent non-specific bystander B-cell activation) [10–14, 23], was missing in all patients with pattern II or III lesions tested. Some 91% of the pattern II and III MS samples did not even show a monospecific IgG response to one of the constituents of the MRZR; in contrast, a reaction to at least one of the three viral agents has been found in 89–94% of MS patients [14, 19]. Together, this strongly suggests that patients with pattern II and pattern III lesions are immunopathophysiologically distinct from patients with ‘pattern I MS’ as well as from previous MS cohorts [8, 10, 19].

**Fig. 3:**
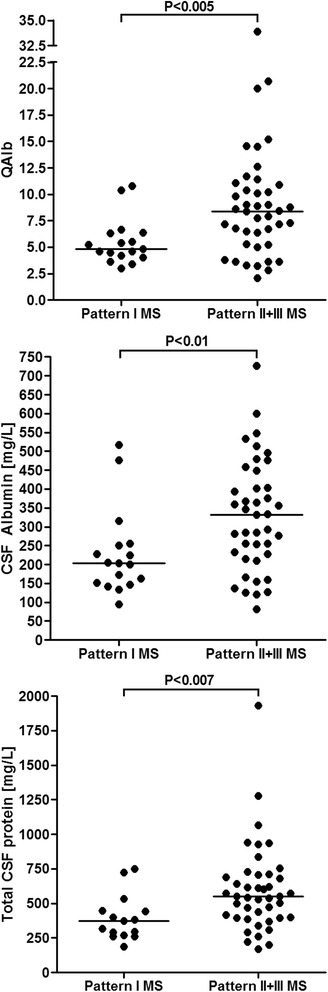
Albumin CSF/serum ratios, CSF albumin concentrations, and CSF total protein concentrations. *QAlb* CSF/serum albumin ratio

This notion is further supported by the finding of additional, significant differences in CSF profiles between patients with pattern I lesions and patients with pattern II or III lesions. The latter group significantly more frequently had signs of compromised blood–CSF barrier function, had significantly higher CSF albumin concentrations and significantly higher and more frequently elevated CSF total protein levels (Tables 6 and 9; Fig. 3). In addition, an albuminocytological dissociation was more commonly seen in patients with type II or III lesions.

Although intraindividual persistence of a single lesion type over time has been shown in a larger cohort of biopsied patients [4, 5], there is an ongoing discussion on whether the various histopathological lesion patterns identified in MS really represent different entities or rather different stages of the same disease. Given that OCBs are a constant feature in MS, which once acquired does not normally vanish [24, 25] (except in rare patients treated with natalizumab [26, 27], a drug not used in any of our patients), the lack of OCB positivity or persistence in most pattern II and III patients provides particularly strong evidence in favour of the former hypothesis.

The term ‘multiple sclerosis’ refers to a clinicoradiologically defined syndrome. There is currently no proof that all patients presenting with acute CNS demyelination and dissemination in time and space share the same underlying pathogenesis. Instead, several lines of evidence suggest that the current ‘phenotypic’ definition of MS may cover more than one disease: (i) pathological studies have demonstrated histopathological heterogeneity among patients with MS [1, 4, 5]; (ii) clinical studies have shown differences in clinical presentation (‘spinal MS’), course (primary progressive MS without relapses vs. relapsing-remitting MS), severity and prognosis (‘benign MS’); (iii) MRI studies have suggested different lesion types in MS; and (iv) treatment trials have found ‘non-responders’, i.e. patients in whom drugs shown to be effective in the majority of patients with MS were of no benefit. In accordance with that hypothesis, numerous patients previously diagnosed as having variants of MS or ‘opticospinal MS’ based on the current clinicoradiological consensus criteria were found over the past decade to have newly discovered humorally mediated diseases, pathophysiologically distinct from MS, that are now termed AQP4-IgG-positive ‘neuromyelitis optica spectrum’ disorder (NMOSD) [28–33] and MOG-IgG-positive encephalomyelitis (EM) [17, 34–42]. Of note, many of patients with AQP4-IgG-positive NMO or MOG-IgG-positive EM had previously been wrongly diagnosed with MS due to a significant overlap in clinical presentation and clinical criteria and, in consequence, had been wrongly treated with drugs approved for MS but not for NMO or MOG-EM [17, 34–36, 43–47]. In a large European cohort, more than 40% of AQP4-IgG-positive patients with NMOSD had been previously misdiagnosed with MS [43]. Similarly, McDonald’s clinicoradiological criteria or Barkhofs’s radiological for MS were met by 33 and 15%, respectively, of all MOG-IgG-positive patients in a recent study [35]. Wrong diagnosis and, accordingly, false treatment for MS may have caused disease exacerbation and worse outcome in some of those patients, underlining the clinical importance of studies investigating potential heterogeneity in MS [45–47].

AQP4-IgG-positive NMO lesions, MOG-IgG-positive EM lesions and pattern II MS lesions share important histopathological similarities in that all three are characterized by antibody and complement deposits. It is therefore of particular note that many of the CSF findings from patients with pattern II lesions were more similar to what has been reported in AQP4-IgG-positive NMO [43, 48, 49] and MOG-IgG-positive EM [17, 34–36] than to what was found in patients with pattern I lesions in our study (Tables 1, 2, 3, 4, 5, 6, 7, and 8). However, the majority of patients with pattern II and pattern III lesions are negative for AQP4-IgG and MOG-IgG, as were all 10 patients with pattern II or III lesions and available serum samples in the present study [18, 50, 51], suggesting a potential role of other, so far unknown autoantibodies in this condition. In contrast, the CSF findings associated with pattern I lesions were much more in line with what one would expect in patients with typical MS.

Of interest, AQP4-IgG-positive NMO is characterized by a marked female predominance (male to female ratio ~1:10–12) [43]; in contrast, the male to female ratio was 1:2.2 in the pattern II patients and thus more similar to what has been reported in MS and in MOG-IgG-positive EM [35].

In Germany, ≥95% of all MS patients are positive for CSF-restricted OCB [8, 10, 19]. The fact that most pattern II and III patients had no CSF-restricted OCB in our study therefore implies that pattern II and III MS cannot account for the majority of patients with MS. With most pattern II and III patients belonging to the small subgroup of <5% of OCB-negative MS cases, pattern II MS and pattern III MS are probably rare conditions.

This seems to be in contrast to histopathological studies that reported a lower proportion of pattern I cases. However, there are several reasons why pattern II and III cases may be overrepresented in neuropathological cohorts:

- As OCBs are considered a hallmark of MS, it seems reasonable to conceive that patients with clinical and radiological findings suggestive of MS but no OCB are more likely to undergo brain biopsy or autopsy than those with OCB. The lack of intrathecal IgG synthesis in patients with pattern II or pattern III lesions may thus result in overrepresentation of pattern II and pattern III patients in histopathological studies.
- Tumefactive lesions are more commonly found in pattern II and III patients and certainly tend to prompt biopsies and autopsies more often than conventional lesions. Similarly, most pattern II and III patients in autopsy studies had died from highly active disease (in particular, patients with pattern III lesions, who often had died from fulminant disease within a few months from onset). However, fulminant and highly active disease definitely also ﻿t﻿e﻿nd to prompt biopsy and autopsy. This may again result in an overrepresentation of pattern II/III patients in biopsy/autopsy studies.
- Some patients with pattern II lesions are positive for MOG-IgG [4, 18, 52]. MOG encephalomyelitis shows a strong clinical and paraclinical overlap with MS but also significant differences such as ADEM-like presentation, longitudinally extensive and bilateral ON, longitudinally extensive myelitis [17, 34–36]. Again, such ‘MS-atypical’ presentations may tend to prompt biopsy or autopsy and, thus, artificially increase the proportion of pattern II cases in biopsy/autopsy studies.

Our results indicate that the proportion of patients with type II and type III lesions may be particularly high among OCB-negative patients diagnosed with MS. While only 2–10% of patients with MS are negative for OCB according to the literature, this subset is not small in absolute numbers, given the high prevalence of MS in some Western populations (e.g. ~2.3 million patients worldwide; ~140,000 patients in Germany). Given that fact and considering that differences in pathophysiology may well translate into different treatment requirements, as already shown in AQP4-IgG-positive NMO [47] and suggested for MOG-IgG-positive EM [34, 35], studies aiming at enhancing our understanding of the immunopathophysiology of pattern II and III lesions seem highly warranted.

Importantly, the frequency of OCB has been reported to increase with latitude [53, 54]. Whether this implies a higher proportion of patients with ‘pattern I’ lesions at higher latitudes is currently unknown but deserves to be addressed in future studies.

We acknowledge that our study has both strengths and limitations. While we count the high number of CSF samples included (*n* = 68) and the high number of CSF parameters analysed among the strengths of our study, the retrospective and exploratory study design is a potential limitation. However, prospective studies would be extremely difficult or even impossible to perform given that brain biopsies are only very rarely performed in patients with MS and that lumbar puncture is not anymore required to make the diagnosis of MS according to the current international diagnostic criteria [3].

## Conclusions

In summary, the present study provides additional, strong evidence for pattern I MS on the one hand and pattern II MS and pattern III MS on the other hand being distinct entities by demonstrating significant differences in CSF findings between patients with pattern I lesions and patients with pattern II or pattern III lesions, especially with regard to intrathecal IgG synthesis. Of particular note, the CSF profiles present in the pattern II/III subgroup were more similar to those reported in AQP4-IgG-positive NMO and MOG-IgG-positive EM than to those classically considered typical for MS. Further studies are now warranted to confirm our findings in larger, international cohorts. Such studies need to take into account the recently reported increase in frequency of CSF oligoclonal banding in MS with latitude [53, 54].

## Abbreviations

AQP4: Aquaporin-4
CNS: Central nervous system
CSF: Cerebrospinal fluid
EM: Encephalomyelitis
IgA: Immunoglobulin A
IgG: Immunoglobulin G
IgM: Immunoglobulin M
MOG: Myelin oligodendrocyte glycoprotein
MRZR: Measles, rubella, zoster reaction
MS: Multiple sclerosis
NMO: Neuromyelitis optica
NMOSD: NMO spectrum disorder
OCBs: Oligoclonal bands
QAlb: Albumin CSF/serum ratio
QIgG/A/M: IgG/A/M CSF/serum ratio
TP: Total protein
TPHA: *Treponema pallidum* haemagglutination assay
WCC: White cell count
